# Testicular and inguinal lymph node metastases of medullary thyroid cancer: a case report and review of the literature

**DOI:** 10.1186/1472-6823-14-84

**Published:** 2014-10-11

**Authors:** Marialuisa Appetecchia, Agnese Barnabei, Vincenzo Pompeo, Steno Sentinelli, Roberto Baldelli, Salvatore Maria Corsello, Francesco Torino

**Affiliations:** Regina Elena National Cancer Institute, Endocrinology Unit, Via Elio Chianesi 53, I- 00144 Rome, Italy; Department of Urology, Regina Elena National Cancer Institute, Via Elio Chianesi 53, Rome, Italy; Department of Pathology, Regina Elena National Cancer Institute, Via Elio Chianesi 53, Rome, Italy; Endocrinology Unit, Università Cattolica, Largo F. Vito, 8, 00168 Rome, Italy; Department of Systems Medicine, Chair of Medical Oncology, Tor Vergata University of Rome, Via Montpellier, 1, 00133 Rome, Italy

**Keywords:** Medullary thyroid cancer, Testicular metastasis, Inguinal lymph node metastasis

## Abstract

**Background:**

The involvement of the testis by metastatic medullary thyroid carcinoma has never been described before. We describe the first case of metastatic medullary thyroid carcinoma affecting testis and inguinal lymph nodes.

**Case presentation:**

A 73-year-old Caucasian man was referred to undergo urologic surgery due to a painless nodule in the right testis and an homolateral inguinal lymphoadenomegaly. The patient had a history of medullary thyroid carcinoma with relapsing disease to the spine and lung nodules. Serum calcitonin and CEA levels were 175 pg/ml and 22 ng/ml, respectively. With suspected testicular cancer, the patient underwent radical right orchiectomy with the excision biopsy of the right inguinal lymph node. Histopathology and immunohistochemistry revealed that both the lesions were due to metastases from medullary thyroid carcinoma.

**Conclusion:**

Metastases to the testis and inguinal lymph nodes may be due to various solid and hematological tumors. This case, despite its rarity, suggests that testis and inguinal lymph nodes should be considered as potential secondary sites of medullary thyroid carcinoma as well.

## Background

Medullary thyroid carcinoma (MTC) is a neuroendocrine tumor that arises from the thyroid parafollicolar calcitonin producing C cells and accounts for about 5% of thyroid carcinomas [1]. MTC mainly presents as a sporadic disease, but in approximately 20% of cases is hereditary. Specific germline mutations in the rearranged during transfection proto-oncogene (*RET*) have a pathogenic role in the onset of familial MTC (FMTC) or MEN2, the two clinical forms of hereditary MTC [2–4]. In these patients, *RET* mutations predict prognosis and guide the choice of treatment timing and follow-up. However, genetic testing for germline *RET* mutations is recommended not only for screening children and adults in known kindreds with inherited forms of MTC, but also for all newly diagnosed patients with clinically apparent sporadic MTC [1, 2].

Sporadic MTC usually manifests in the fifth-sixth decade, while familial forms have an earlier onset [1]. MTC usually presents as a palpable neck mass due to thyroid nodule(s) and in 30-50% of cases it is accompanied by metastases in cervical/paratracheal lymph nodes. Upper and anterior mediastinal lymph nodes are also in the pathway of tumor spread, but symptoms related to aerodigestive tract compression/invasion are reported by up to 15% of patients [5]. Distant metastases are present at the diagnosis in 10-15% of patients. Preferred sites of metastatic spread include lung, liver and bone [6]. Metastases to brain, adrenal glands, pleura, heart, ovary, pancreas, pituitary, retina, skin and breast have been rarely/exceptionally reported [7–9].

Compared with papillary/follicular thyroid cancer, MTC is more aggressive having a higher rate of recurrence and increased mortality [10]. Older age, larger tumor size, involvement of regional lymph nodes and distant metastases correlate with worse prognosis as well as high calcitonin and carcinoembrionic antigen (CEA) serum levels [11]. The clinical course of advanced/metastatic MTC is unpredictable. Lung or bone metastases may initially cause symptoms in only 5-10% of patients. Survival in patients with newly diagnosed distant metastases is 51% at 1 year, 26% at 5 years and 10% at 10 years [10, 12, 13].

Surgery is the main treatment for primary MTC and for local and distant metastases, whenever feasible. External-beam radiation to the neck/upper mediastinum is useful in patients with extrathyroidal disease or extensive nodal metastases not undergoing curative resection and for palliative purposes [2]. Cytotoxic chemotherapy is used in patients with metastatic/unresectable MTC, but is poorly effective [6]. Recently, vandetanib and cabozantinib, two oral multikinase inhibitors improved survival in patients with advanced/metastatic MTC and are licensed for the treatment of these patients [14]. Several experimental drugs, mainly kinase inhibitors, are under clinical evaluation [15, 16].

Herein, we report the case of a patient with MTC that metastasized to the right testis and to an homolateral inguinal lymph node, many years later to the detection of skeletal and lung metastases.

## Case presentation

In 2002, a 63 year old Caucasian man presenting a large solitary nodule in the upper right lobe of thyroid was cytologically diagnosed with medullary thyroid carcinoma (MTC). No family history of MTC or multiple endocrine neoplasia (MEN) was reported. Appropriate endocrine work up excluded MEN2. Preoperative serum calcitonin was 122 pg/ml (reference range: 0-9.6 pg/ml) with normal carcinoembryonic antigen (CEA) serum levels. The patient underwent total thyroidectomy and bilateral neck lymph node dissection. Histology confirmed a 52 mm in diameter MTC confined to thyroid without metastases in the 54 resected lymph nodes (stage III; UICC 2002). The genetic testing identified the non-conservative functional c.2071G > A polymorphism in codon 691 of the RET proto-oncogene that was not mutated.

During the follow-up calcitonin (basal and after pentagastrin stimulation) and CEA were in the normal range until September 2008, when basal calcitonin started to slightly increase (up to 64 pg/ml). Neck ultrasound was negative, but contrast-enhanced computed tomography scan (CT-scan) showed diffused lung micronodules. Then, the 99mTc-MDP bone scan showed an uptake area at the pedicle and right lamina of the fifth lumbar vertebra (November 2009) and 18-Fluorodeoxyglucose Positron Emission Tomography (^18^FDG-PET) and ^68^Ga-DOTATOC-PET confirmed the fifth lumbar vertebra as the only metastatic site. In 2011, the disease progressed (at bone scan the known uptake area enlarged and a new pathologic area appeared in the right hemisacrum). Bone lesions were osteoblastic on X-ray evaluation. Lung metastases remained stable in the absence of new lesions at CT-scan. Serum calcitonin and CEA levels were 141 pg/ml and 21 ng/ml, respectively. The patient was still asymptomatic.

**Figure 1:**
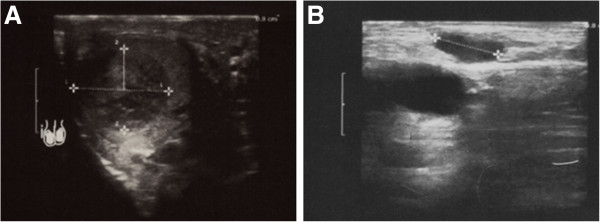
**Ultrasound images show a solid and inhomogeneous hypoechoic mass in the upper lobe of the right testis (A) and a right inguinal lymphoadenopathy 2 cm in diameter (B).**

In March 2012, the patient presented with a painless nodule in the right testis and a palpable right inguinal lymph node. Ultrasound showed an hypoechoic and inhomogenous solid mass of 17 mm in size in the upper lobe of the right testis and a 2 cm inguinal lymph node (Figure 1). β-human chorionic gonadotropin (β-HCG), α-fetoprotein and lactate dehydrogenase were normal. Calcitonin was 175 pg/ml, CEA 22 ng/ml. CT-scan showed a slight increase in two lung nodules (each 1 cm in diameter).

**Figure 2:**
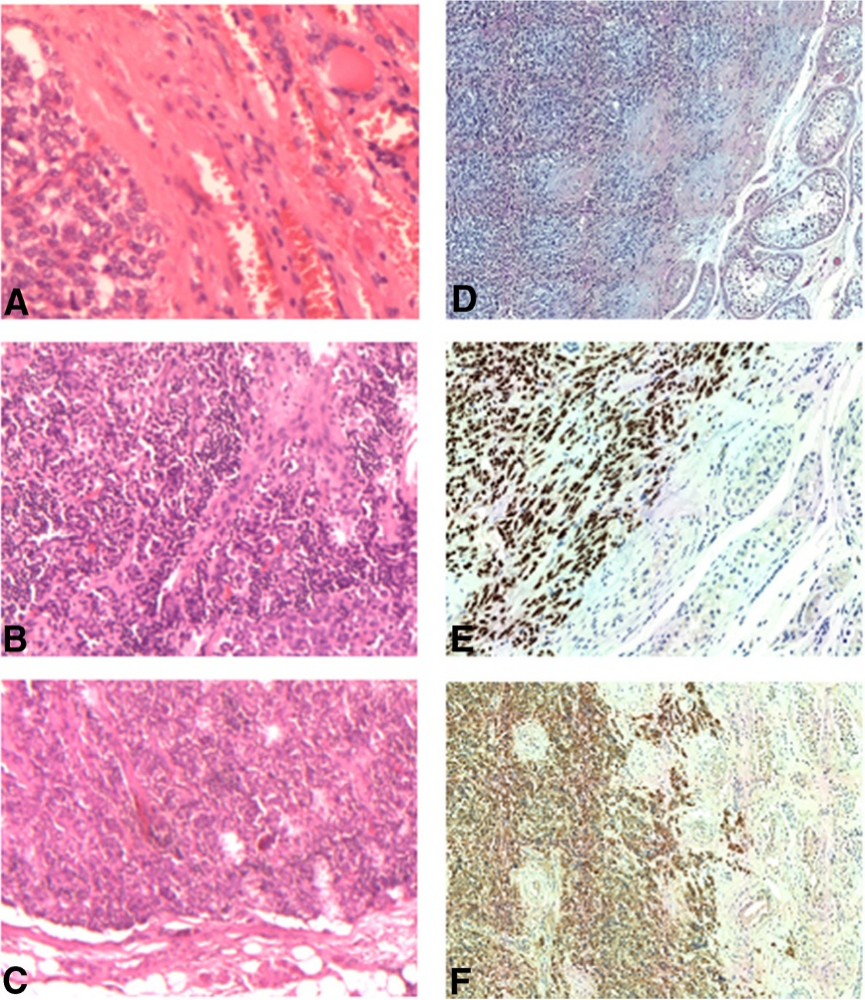
**Histological and immunohistochemical examinations. A**, **B**: Histology of primary medullary thyroid cancer; **C**: Metastasis of medullary thyroid carcinoma in the right inguinal lymph node; **D**: The right testis: histological examination shows trabecular solid areas with round and spindle cells of medullary thyroid carcinoma, partially separated by fibrous stroma infiltrating testicular parenchyma. Immunohistochemistry: Tumor cells show diffuse cytoplasmic positivity for TTF-1 **(E)** and with calcitonin **(F)**.

Given that primary testicular cancer was suspected, the patient underwent right radical orchiectomy and excision of the inguinal lymph node. Histology revealed the presence of metastases, morphologically resembling MTC, both in the testis (2.2 cm in diameter) where tumor cells infiltrated the *rete testis*, and in the inguinal lymph node. Immunohistochemistry showed reactivity for AE1/AE3, TTF-1 calcitonin and chromogranin, but not for CD30, actin-μ and vimentin, thus confirming the diagnosis of metastases from MTC (Figure 2).

Post-surgery calcitonin and CEA levels decreased, but did not normalize. Recently, progressive disease was documented in the lung, bone and inguinal lymph nodes. The patient is still asymptomatic, but is going to start vandetanib.

## Discussion

Testicular metastases are extremely rare, except in patients affected by leukemia or lymphoma [17]. Metastases to the testis from solid tumors may be discovered incidentally at autopsy or following therapeutic/diagnostic orchiectomy, as in the case presented. In autopsy series that included non-neoplastic deaths, testicular metastases were found in 0.02% to 2.5% of specimens [17–21]. In other series of patients who underwent orchiectomy for testicular malignancy, testicular metastases were detected in up to 7% of patients, the common primary malignancy being prostate, melanoma, sarcoma, lung, urologic and gastrointestinal cancer (Table 1) [22–27]. Less frequently urinary bladder and pancreatic tumors or neuroblastoma metastasize to the testis [28]. Bilateral involvement of the testis occurs in about 15% of cases [17–27]. Involvement of the testis is usually accompanied by cancer spreading to other sites, rarely affecting survival that remains largely dependent on the burden and aggressiveness of the underlying disease and the vital importance of involved organs. Surgery, if indicated, is the preferred therapy of testicular metastases from solid malignancies. Testicular metastasis from MTC has never been reported.

**Table 1:** **Selected case series reporting metastases to the testis**

Authors	Diagnostic/therapeutic/autopsy series	Total number of patients included	N. of patients with testicular metastases (%)	Primary solid tumors (%)
Pienkos et al. [20]	Autopsy	24,000	15 (0.06)	Lung 7 (46.6); Melanoma 2 (13.2); Ureter 1 (6.7); Kidney 1 (6.7); Rectum 1 (6.7); Salivary gland 1 (6.7); Pancreas 1 (6.7); Unknown 1 (6.7).
Nistal et al. [28]	Autopsy	3,474	5 (0.14)	Pancreatic 1 (20); urinary bladder 1 (20); prostate 1 (20); neuroblastoma 2 (40).
Tiltman AJ. [18]	Autopsy	2,271	6 (2.5)	Prostate 2 (33.3); melanoma 2 (33.3); lung 1 (16.7); pleural mesothelioma 1 (16.7)
García-González et al. [21]	Autopsy	780	5 (0.64)	Lung 3 (60); melanoma 1 (20); pancreatic endocrine carcinoma 1 (20).
Patel et al. [23]	Autopsy/diagnostic/therapeutic	550	20 (3.6)	Prostate 12 (60), melanoma 3 (15), sarcoma 2 (10), lung 1 (5), colon 1 (5), renal carcinoma 1 (5)
Sharma et al. [25]	Diagnostic/therapeutic	300	10 (3.33)	Prostate 6 (60); lung 1 (10); larynx 1 (10); kidney 1 (10); colon (10).
Lu et al. [26]	Diagnostic	200	14 (7)	Prostate 8 (57.1); Lung 2 (14.3); Esophagus 1 (7.1); Seminal vescicles 1 (7.1); Sigmoid colon 1 (7.1); Unknown 1 (7.1).
Dutt et al. [19]	Autopsy/diagnostic/therapeutic	31	22	Prostate 6 (27.4); stomach 5 (22.8); lung 3 (13.6); melanoma 2 (9.2); neuroblastoma 1 (4.5), medulloblastoma 1 (4.5); rectum 1 (4.5); rhabdomyosarcoma 1 (4.5); pancreas 1 (4.5); unknown (4.5) (leukemias: 9 cases).
Ulbright et al. [27]	Diagnostic	26	26	Prostate 11 (42.3); renal 4 (15.4); colon 4 (15.4); urinary tract 3 (11.5); lung 2 (7.7); esophagus 1 (3.8); small intestine 1 (3.9).
Grignon et al. [22]	Diagnostic/autopsy	18	18	Lung 9 (50), prostate 4 (22.2), stomach 4 (22.2), bile tract 1 (5.6).
Bhasin et al. [24]	Diagnostic/therapeutic	10	10	Prostate 4 (40); gastrointestinal 4 (40); kidney 2 (20).

The mechanisms of metastatic spread to the testis is still speculative. It has been suggested that metastases from solid tumors might join testis via retrograde arterial, venous or lymphatic embolization [29]. Specific spreading routes have been associated with certain solid tumors, including arterial embolism from lung cancer [30], retrograde venous spread in renal cell carcinoma [29] or retrograde lymphatic spread in gastrointestinal, prostate and urinary bladder cancer [28, 29, 31]. In an anatomic study of spermatic veins, Wishami *et al.* demonstrated connections between spermatic and renal capsular veins and between spermatic and ipsilateral colonic veins. These findings may sustain the retrograde venous spread from both kidney and colon cancer [32]. Moreover, the absence of valves in spermatic veins has been suggested as a favoring factor for the retrograde spread from the renal veins or the retroperitoneum [31]. Metastases from prostatic carcinoma and seminal vesicle cancer may reach testis via direct extension and intraductal spread via the vas deferens and epididymis [22, 23].

In patients presenting a solitary testicular mass, the differential diagnosis must include metastatic carcinoma. A personal history of cancer should first be investigated. Older age is suggestive of a secondary testicular tumor. Measurement of serum α-fetoprotein and β-HCG, if negative, does not exclude a testicular primary germ cell tumor. However, post-surgery/-biopsy histopathology evaluation is almost invariably required to address the differential diagnosis of a testicular mass [33].

Histology features of testicular metastases are quite different from primary tumors of the testis. The former present as a nodule of small nests of tumor cells more commonly located in the epididymis and para-epididymal area, with a widespread involvement in the interstitium and the relative sparing of the seminiferous tubules [17]. Interstitial cell tumors or early invasive seminoma may present similar features [17]. However, the absence of seminiferous tubular involvement is a distinguishing aspect of metastatic lesions [17, 23]. Extensive vascular and lymphatic invasion of parenchyma and tunica albuginea, multifocality or bilaterality are also features suggestive of metastases from solid malignancies. The immunostaining positivity for epithelial membrane antigen (EMA) and placental alkaline phosphatase negativity are consistent with a secondary tumor [26]. Similarly, the absence of EMA-positive immature testicular germ cells are also features favoring metastasis rather than primary testicular tumors [26].

Even though, the rarity of metastatic involvement of testis remains unexplained, it may be due to blood-testis and blood-epididymis barrier [34, 35]. The tight junctions between Sertoli cells and between epididymis epithelial cells respectively, build a functional barrier of utmost competence [36]. These barriers create a unique anatomical, physiological and immunological microenvironment that restrict the passage of molecules and cells from entering or exiting the lumen [35, 36]. This condition allows the proper development of the germ cells into fully functional sperm, most likely limiting metastatic deposits as well [35, 36]. It has also been suggested that low temperature in the scrotum creates an unfavorable environment for the seeding of metastases [17, 34–37]. However, molecular mechanisms allowing the blood-testis and blood-epididymis barriers to prevent metastatic spread to testis and how metastases overcome these protective mechanisms remain still unclear.

Inguinal lymph nodes are not a known site of metastatic spread from MTC. Metastases to inguinal lymph nodes are more commonly involved by cells from lymphoma and other malignant tumors located in the skin of lower extremities, in the scrotum, penis, vulva, clitoris, lower third of the vagina, anal canal, and infraumbilical region of the anterior abdominal wall. Melanoma or squamous cell carcinoma arising on the skin of the trunk may also metastasize to the inguinal lymph nodes. Rarely prostate, testicular or colon primary malignancies metastasize to inguinal lymph nodes [38, 39]. When this happens, previous inguinal, pelvic or scrotal surgery may have altered lymphatic drainage [40]. Injured lymphatics may harbor new anastomoses between testicular and inguinal/pelvic lymphatic plexes, providing a potential direct way of metastatic spread to the inguinal lymph nodes [41]. Alternatively, the involvement of the adjacent anterior abdominal wall by micrometastasis or preexisting aberrant lymphatic drainage route have been postulated [41].

It is presumable that MTC metastases reached the testis first and then the inguinal lymph node via the infiltration of the *rete testis*. However, it is impossible to establish whether metastatic spread occurred *vice versa* or simultaneously.

Another interesting aspect of the present case, lies in the unexpectedly low levels of serum calcitonin and CEA in relation to the widespread dissemination of the disease. Calcitonin is a specific serum marker of MTC [1]. Preoperative calcitonin levels are very useful for both diagnosis and staging [1, 2]. In case of nodal metastases, basal calcitonin levels commonly range between 10-40 ng/ml (normal range, <10 ng/ml), whereas distant metastases are usually associated with a calcitonin level >150 ng/ml and often >1000 ng/ml [6, 16]. Moreover, CEA may be increased in more than 50% of MTC patients and may be useful especially when preoperative calcitonin is within normal range [16]. A preoperative CEA serum level >30 ng/ml predicts poor prognosis and indicates that surgery may not be curative. CEA levels >100 ng/ml suggest extensive lymph node involvement and distant metastases. Increasing CEA levels with stable calcitonin suggest tumor dedifferentiation and worse prognosis [6, 16]. Serum calcitonin and CEA measurements 2-6 months after the surgery are recommended to detect residual disease. Patients with undetectable serum calcitonin (basal and after stimulation test) and normal CEA values are virtually cured, being approximately 3-5% their 5-year recurrence rate [6, 13]. High basal serum calcitonin values ≥6 months after surgery are suggestive of residual disease. Moreover, calcitonin and CEA doubling times strongly predict disease progression and reduced survival [42, 43]. In our opinion, the low serum levels of calcitonin and CEA (175 pg/ml and 22 ng/ml, respectively), despite lung, bone, testicular and inguinal lymph node metastases, are consistent with the low aggressiveness and the smoldering progression of the disease. These biological features may be not rarely seen in patients with MTC.

## Conclusions

To the best of our knowledge, this is the first case report on testicular metastasis of MTC. Also inguinal lymph node involvement as a site of relapsing MTC has never been described before. In this patient, MTC metastases to the testis and inguinal lymph node were associated with disseminated disease, which is commonly observed in testicular metastases from solid malignancies. The present report, beyond its rarity, emphasizes that physicians need to consider testis and inguinal lymph nodes as an unusual, but possible site of metastases from MTC, even in the presence of relatively low serum markers of malignancy.

## Consent

Written informed consent was obtained from the patient for publication of this Case report and any accompanying images. A copy of the written consent is available for review.

## Abbreviations

β-HCG: β-human chorionic gonadotropin
CEA: Carcinoembrionic antigen
CT: Computed tomography
FDG-PET: 18-Fluorodeoxyglucose Positron Emission Tomography
EMA: Epithelial membrane antigen
FMTC: Familial medullary thyroid cancer
MTC: Medullary thyroid cancer
MEN: Multiple endocrine neoplasia
RET: Rearranged during transfection proto-oncogene
TTF-1: Thyroid transcription factor-1.
